# Understanding CAM Natural Health Products: Implications of Use Among Cancer Patients and Survivors

**DOI:** 10.6004/jadpro.2013.4.5.2

**Published:** 2013-09-01

**Authors:** Judith M. Fouladbakhsh, Lynda Balneaves, Elizabeth Jenuwine

**Affiliations:** From College of Nursing, Wayne State University, Detroit, Michigan; School of Nursing, University of British Columbia, Vancouver, British Columbia, Canada

## Abstract

Herbs, vitamins, and other natural health products are being used by cancer patients and survivors with increasing prevalence in the United States. These complementary and alternative medicine (CAM) products, which are also referred to as natural health products in Canada and abroad, are used during cancer treatment and the survivorship period to ease the burden of symptoms such as pain, fatigue, insomnia, anxiety, and depression and hence improve overall quality of life. Data indicate that while patients choose these products for self-treatment, they often do not inform their health-care providers, thereby presenting the potential for negative interactions. This article gives an overview of CAM natural health products, including discussion of herbs, vitamins, and other supplements such as minerals, enzymes, and more. Related research is presented, and implications for advanced practitioners are discussed. Insights into guiding safe and effective use among patients as well as appropriate decision-making strategies are explored.

The use of herbs, vitamins, and other complementary and alternative natural health products continues to be highly prevalent in the United States, particularly among individuals of varying ages who have been diagnosed with cancer and other chronic illnesses (Bright-Gbebry et al., 2011; Fouladbakhsh & Stommel, 2008; Gratus et al., 2009; Greenlee et al., 2009; Miller et al., 2008; Post-White, Fitzgerald, Hageness, & Sencer, 2009). These natural products are often used by cancer patients to promote health, enhance the treatment of illness and ease side effects, prevent cancer recurrence, strengthen immunity, and improve mood and quality of life through the management of burdensome and persistent symptoms (Astin, Reilly, Perkins, & Child, 2006; Deng & Cassileth, 2005; Fouladbakhsh & Stommel, 2008, 2010; Post-White et al., 2009; Verhoef, Balneaves, Boon, & Vroegindewey, 2005; Wells et al., 2007). Given the availability and high prevalence of natural health products for self-treatment, it is imperative that advanced practitioners understand the complexity of these products, the decision-making process, and the implications of their use across the cancer trajectory.

This article provides an overview of natural health products found within CAM, describing mechanisms of action, interaction with conventional treatments, and the potential benefits and risks. Guidelines to maximize beneficial patient outcomes and minimize harmful interactions are presented along with an overview of the recent research literature

## THE ROLE OF NATURAL HEALTH PRODUCTS

**THEORETICAL PERSPECTIVES**

The world of CAM is extensive and diverse, incorporating a wide array of therapies that include provider services, practices, and products, many of which are nested within whole systems of health care across the globe. These systems of care, often referred to as alternative medicine and more recently referred to as whole systems of care, have historical and philosophical roots that often extend over millennia. Most include different perspectives and beliefs about health, illness, treatment, and ways of living that influence wellness, recovery, and the birthing and dying processes.

The umbrella term "CAM" includes thousands of diverse medical and health-care treatments, services, products, and practices that are not considered part of conventional western biomedicine. The National Center for Complementary and Alternative Medicine (NCCAM) has categorized CAM therapies as follows: (a) whole systems of alternative health care such as traditional Chinese and ayurvedic medicine; (b) mind-body therapies such as yoga and tai chi; (c) manipulative and body-based approaches such as massage and chiropractic; (d) energy therapies such as Reiki and Healing Touch; and (e) natural and biologically based products that include herbs, special diets, vitamins, essential oils, and other botanical supplements (NCCAM, 2008).

In contrast to the NCCAM categorization, the CAM Healthcare Model views CAM from a health service utilization perspective, allowing one to examine use of CAM providers, CAM practices and/or CAM products, either as separate categories or in combination, which is the most prevalent pattern of use in the United States (Fouladbakhsh, 2010; Fouladbakhsh & Stommel, 2007, 2008, 2010). The CAM Healthcare Model allows inclusion of the philosophical and theoretical foundations related to specific therapies, including many products, that influence the decision for CAM use and may potentially affect health outcomes in diverse patient populations. Serving as a framework for this article, the model also highlights the importance of considering attitudes and beliefs about natural health products, in particular the prevailing view that because something is "natural" it is automatically beneficial and without harm. The power and incredible complexity of natural products should not be underestimated, but rather intensely studied to ascertain potential benefits and curative/healing effects along with potential risks and interactions with conventional treatments.

As evidence accumulates to support beneficial outcomes for herbs, vitamin supplements, and other products, their inclusion in conventional care is increasing. This trend toward integrative practice whereby evidence-based CAM is incorporated into conventional health care increases options for treatment, symptom management, and improved quality of life. It also presents advanced practitioners with the challenge of assessing CAM use by their patients and understanding these products’ effect on health and response to conventional cancer treatment.

A holistic perspective to health and wellness, a foundation within nursing, is also receiving renewed interest, with an emphasis on the important interrelationships of the body, mind, and spirit to enhance healing of the whole person from birth to death (Dossey, Keegan, & Guzzetta, 2000). Inherent within holistic philosophy is the emphasis on self-care, which resonates with the use of CAM products for health promotion, wellness and illness prevention, and treatment. This orientation toward holism, as well as the extensive training of advanced practitioners with regard to assessment, diagnosis, and care, makes them particularly well suited to addressing the use of these products and the associated decisions made by patients.

**PREVALENCE AND PURPOSE OF CAM PRODUCT USE IN THE CANCER POPULATION**

Over the past decade, the use of CAM therapies, which include innumerable botanical and natural products, has increased among the US cancer population, with almost 44% reporting use of a CAM therapy in 2007 as compared with 37% to 40% in 2002, based on analysis of the National Health Interview Survey (NHIS) data (Fouladbakhsh, 2010; Fouladbakhsh & Stommel, 2008; Mao, Farrar, Xie, Bowman, & Armstrong, 2007). Of the many therapies and practices inherent within CAM, the use of vitamins and herbs often ranks highest and is significantly higher (* p* < .001) among those who have experienced cancer (66.6% and 22.7%, respectively) compared with those who have not (Fouladbakhsh, 2010; Fouladbakhsh & Stommel, 2008).

Estimates of overall CAM use in the United States range from 34% to 76% depending on the therapies included and the population (Barnes, Bloom, & Nahin, 2007; Barnes, Powell-Griner, McFann, & Nahin, 2004; Fouladbakhsh, 2010; Fouladbakhsh & Stommel, 2008, 2010; Mao et al., 2007; Tindle, Davis, Phillips, & Eisenberg, 2005). Use of CAM products only was reported by almost 70% in the cancer population, which is significantly higher than among individuals who never experienced cancer (* p* < .001; Fouladbakhsh, 2010). Use of CAM products was also high among children with cancer, with 59% reporting use within their family (Post-White et al., 2009), and more prevalent among adults with chronic illness (Miller et al., 2008).

Reasons for CAM use include being around other family members who are users of CAM therapies, word-of mouth recommendations, cultural and ethnic traditions, dissatisfaction with conventional treatment, desire for a natural approach, and motivation to try everything (Tindle et al., 2005; Sparber & Wootton, 2001; Vallerand, Fouladbakhsh, & Templin, 2003). Factors that predict use of CAM therapies include gender, age, race, education, chronic illness, and symptom experience (Fouladbakhsh & Stommel, 2008, 2010; Miller et al., 2008; Post-White et al., 2009). Higher CAM use among females, individuals who are middle-aged, and those who are well-educated has also been noted in the general population and among individuals with cancer (Fouladbakhsh, Stommel, Given, & Given, 2005; Tindle et al., 2005).

Symptoms such as pain, insomnia, anxiety, and depression are also strong predictors of CAM use, with variance noted among the types of CAM studied (Fouladbakhsh & Stommel, 2008, 2010). The likelihood of CAM product use was significantly higher among females and individuals with pain and insomnia, whereas being African American or Hispanic resulted in lower odds of use according to data from the NHIS (Fouladbakhsh & Stommel, 2008). In contrast, a lower prevalence of herb use was noted among non-Hispanic Caucasians (Miller et al., 2008) and a high prevalence among African American breast cancer survivors (Bright-Gbebry et al., 2011). The influence of race, ethnicity, and related cultural patterns on CAM use, specifically the use of herb and botanical products, requires further illumination.

Other factors influencing use of CAM therapies include income and health-care provider contact, especially with regard to the vast majority of natural health products that are purchased over the counter in the United States. As shown by studies (Fouladbakhsh, 2010; Fouladbakhsh & Stommel, 2008), the odds of CAM product use almost doubled as income increased; similarly, cancer survivors who were in contact with a nurse practitioner/physician assistant were more likely to use products such as herbs and vitamin supplements. Data have shown that individuals use CAM therapies to promote health and prevent illness such as cancer as well as to enhance cancer treatment and ameliorate side effects.

Among individuals receiving cancer treatment, 37% to 80% reported use of vitamins, herbs, and other CAM products, presenting the potential for complex interactions that are not yet fully understood (Dy et al., 2004; Engdal, Steinsbekk, Klepp, & Nilsen, 2008; Lam et al., 2009; Richardson, Sanders, Palmer, Greisinger, & Singletary, 2000). Products used by children and adults with cancer or other chronic illnesses include multidose and megadose vitamins, a wide range of herbal medicines and natural health products, and other supplements such as minerals (calcium and selenium). Popular products include garlic, ginkgo, green tea, fish oil, tea tree oil, CoQ10, and many other antioxidants.

In sum, the prevalence of CAM use remains high, particularly among cancer patients and survivors of all ages (Fouladbakhsh & Stommel, 2008; Hardy, 2008; Horneber et al., 2012; Mao et al., 2007; Post-White et al., 2009), as do symptom reports of pain, insomnia, fatigue, depression, and anxiety, which may prompt use (Fouladbakhsh & Stommel, 2008, 2010). Complementary and alternative medicine offers additional choices for self-treatment and integrative care, including innumerable natural products available over the counter, online, and through CAM practitioners and integrative health-care practitioners. Understanding the evidence (or lack thereof) with regard to the therapeutic mechanisms, effects, and safety of herbs and other natural health products presents a daunting challenge for nurse practitioners given the extensive array of products, dosing variations, and combined formulations

## MECHANISMS OF ACTION AND INTERACTION

**UNDERSTANDING ACTIONS OF HERBS, VITAMINS, AND OTHER SUPPLEMENTS**

Herbs are complex natural and botanical products that often include multiple ingredients defined as active and inactive molecules and/or compounds that can be used as single or mixed formulations (Cheng, Fan, Ko, Song, & Bian, 2010). Active components can be found in any part of a plant, and herbal medicines may include varying combinations of parts, such as the roots, leaves, berries, blossoms, and/or bark. Forms of herbal preparations include the whole herb, teas, capsules, tablets, extracts and tinctures (alcohol and nonalcohol-based), essential oils, salves, balms, ointments, and lotions. Similarly, vitamins and other supplements may include single or multiple ingredients that are produced, extracted, and/or compounded naturally or synthetically, and can be of varying doses including those in the mega ranges, which far exceed minimum recommended daily requirements.

Herbal products and supplements vary depending upon their action(s) in the body, which may be multifaceted and synergistic (Table 1). These actions are similar to pharmaceutical products, many of which have been developed from herbs and plants, yet other actions are unheard of within a conventional medicine paradigm. For example, adaptogenic herbs are thought to increase resistance to stress and support the adrenal glands, whereas bitters are used to detoxify the liver; these concepts within herbal medicine are organ-supportive and aim to maintain wellness and optimal functioning of specific tissues and organs, thereby preventing disease and enhancing recovery from illness.

**Table 1:**
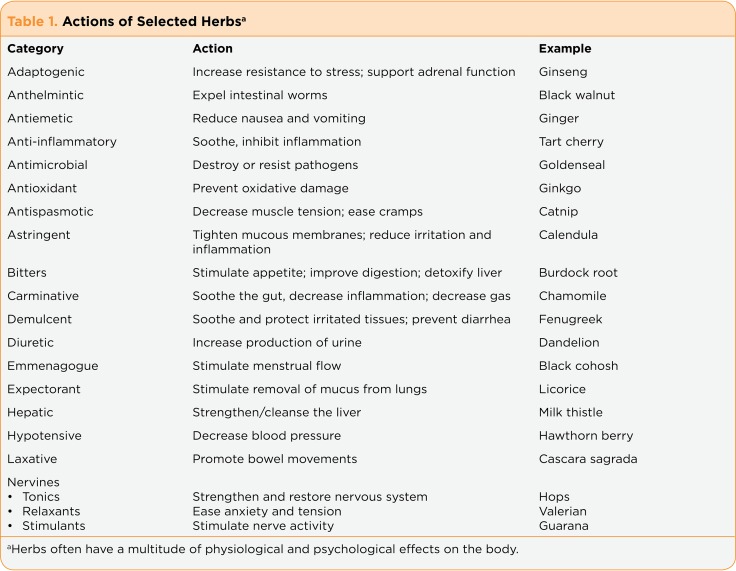
Table 1. Actions of Selected Herbs

As previously mentioned, most of the current information about cancer-fighting activities of medicinal plants and supplements comes from preclinical studies (see Table 21). These studies indicate potential benefits but cannot serve as evidence of these effects in humans. A relatively small number of clinical trials have been published, but they tend to involve few subjects or have methodologic issues. More well-designed large-scale clinical trials are needed to ascertain the benefits and effective dosages of herbal medicines and other natural health products in humans.

**Table 2:**
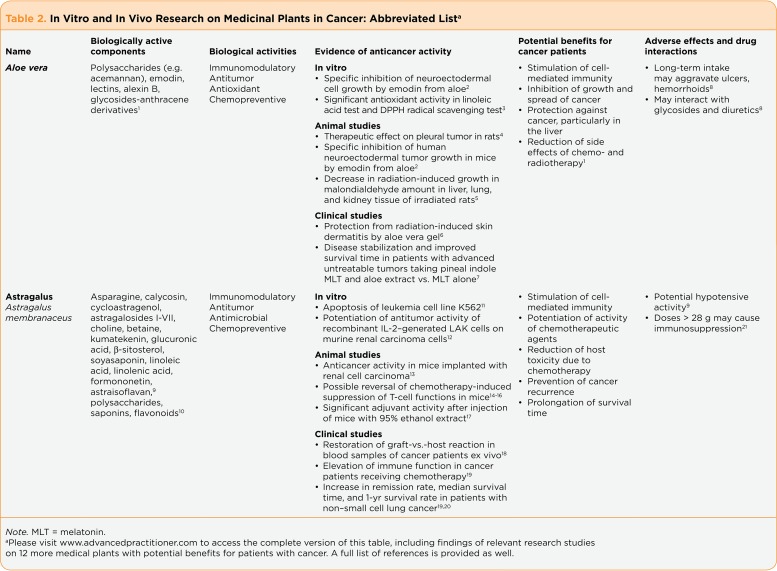
Table 2. Actions of Selected Herbs

The lack of standardization of herbal and other CAM products, either sold to consumers or used in research, poses yet another problem. Very few data are available on the composition and quality of most herbal preparations (Benzie & Wachtel-Galor, 2011). In the United States, herbal medicines are considered dietary supplements and are not regulated by the US Food and Drug Administration (FDA). There can be significant variation in the composition of an herbal medicine depending upon the part of the plant or the extraction method used. In addition, growing conditions—such as the weather during a particular growing season, the geographic area where the plants were grown, or the age of the plants when collected—can affect the composition and the activities of phytochemicals (Benzie & Wachtel-Galor, 2011). Finally, herbal preparations can be contaminated by pesticides or heavy metals or adulterated; for example, PC-SPES, a mixture of several medicinal herbs reported to inhibit androgen-independent prostate cancer, was found to be contaminated with traces of pharmaceuticals and was withdrawn from the market in 2002 (Ben-Arye, Attias, Tadmor, & Schiff, 2010).

Herbal medicines, in particular, are inherently difficult to standardize. A medicinal plant contains many biologically active compounds that can act synergistically, additively, or antagonistically. Concentrations of different compounds may vary depending on the growing conditions and handling. Frequently, individual active compounds are isolated from medicinal plants and their properties are investigated. This practice allows researchers to characterize the biological activities of these individual compounds in vitro and in vivo. It also makes it possible for the results to be replicated by other laboratories or aggregated with other research results in meta-analyses or systematic reviews. This practice of isolating individual active compounds, however, may be difficult and expensive. It also precludes the understanding of possible synergistic effects of multiple biologically active compounds usually present in the same herbal medicine (Benzie & Wachtel-Galor, 2011). Translating this evidence to the whole plant, which is often available in over-the-counter formulations, is difficult and precludes informed consultations regarding the possible effects of herbal medicine.

**NATURAL HEALTH PRODUCT–DRUG INTERACTIONS**

As with drug-drug interactions, the interplay between natural health products and drugs may be defined as pharmaceutical, pharmacokinetic, or pharmacodynamic (Beijnen & Schellens, 2004). Pharmacokinetically, an herb, vitamin, or other natural product taken concurrently with medication may alter absorption, distribution, and/or elimination of a conventional medication. An often-cited example is the effect of St. John’s wort on the bioavailability of antiretrovirals and other medications, which occurs through the herb’s effect on the cytochrome P450 (CYP450) isoenzyme system, the most common system of medication metabolism in the body (Cheng et al., 2010; Hardy, 2008; HemaIswarya & Doble, 2006; Mills et al., 2004; Roby, Anderson, Kantor, Dryer, & Burstein, 2000).

A primary concern during cancer treatment focuses on the effect of natural products on chemotherapy effectiveness. A number of herbal medicines have been shown to modulate the activity of the CYP450 system, which is responsible for metabolism of a large number of prescription medications, including many chemotherapeutic drugs (Block et al., 2008; Hardy, 2008; Lee, 2005). Numerous studies have documented herbal effects that result in an upregulation and others that demonstrate downregulation of medication metabolism, resulting in inhibition and/or stimulation of CYP450 enzymes, thereby altering drug absorption and elimination (Cheng et al., 2010; Hardy, 2008). The inducers of CYP450 enzymes may cause the chemotherapeutic agents to be metabolized more quickly, thus resulting in a lower therapeutic effect or a treatment failure. Conversely, the inhibitors of the CYP450 system may slow down the metabolic clearance of some chemotherapeutic drugs and consequently increase the toxicity of treatment.

St. John’s wort was found to be a potent inducer of the CYP3A4 enzyme, which is responsible for metabolism of many chemotherapy drugs (Hardy, 2008; Lee, 2005). Garlic, ginseng, echinacea, and soy were shown to inhibit several CYP450 isoenzymes in preclinical studies, but human pharmacokinetic studies were either not performed or did not produce measurable changes of chemotherapeutic agents in the blood levels (Hardy, 2008; Lee, 2005). Potential pharmacokinetic interactions between herbs and medications, including chemotherapy drugs, may also occur with adenosine triphosphate binding-cassette transporters such as P-glycoprotein, breast cancer resistance protein, and multidrug resistance-associated protein-1 (Sparreboom, Cox, Acharya, & Figg, 2004), potentially affecting treatment effectiveness due to altered drug metabolism.

Pharmacodynamic interactions influence how the medication and/or natural product affect body tissues and/or organs when used together, resulting in an enhancing (synergistic) or antagonistic effect (HemaIswarya & Doble, 2006). Some herbal medicines may reverse the action of chemotherapeutic drugs and other medications. For example, soy isoflavones may counteract tamoxifen breast cancer prevention therapy, while astragalus and echinacea may reverse cyclophosphamide-induced immune suppression and cause organ rejection in transplant patients (Cassileth, Heitzer, & Wesa, 2009; Cassileth, Yeung, & Gubili, 2008). A number of medicinal herbs also have anticoagulant activity (e.g., ginger, garlic, ginseng, saffron, and turmeric/curcumin) and should be avoided before and after surgery or when taking blood-thinning medications (Cassileth et al., 2009). Finally, there is a concern that taking herbs with strong antioxidant activity may result in protection of tumor cells from oxidative damage during conventional radio- or chemotherapy, leading to treatment failure or a higher likelihood of cancer recurrence (Hardy, 2008).

According to Cheng et al. (2010), herb-drug interactions can be viewed as positive, negative, or neutral, with emphasis needed on promoting safety and expanding clinical research to determine interaction effects. On a positive note, preclinical studies indicate that herbal medicines can "increase the sensitivity of cancer cells to chemotherapeutic drugs, improve survival rates, enhance tumor response to chemotherapeutic drugs, and reduce toxicity of cancer chemotherapy" (Cheng et al., 2010, p. 326). In contrast, negative pharmacokinetic interactions between specific herbs and anticancer drugs have been identified, raising questions about the safety of use during cancer treatment and the effect on tumor survival, although the complexity of effects requires further illumination.

## USE OF HERBAL PRODUCTS IN CANCER PATIENTS

**BENEFITS AND RISKS**

Most cancer patients who choose to take herbs and supplements do so in order to achieve some measure of control over their treatment. They believe that herbal medicines will augment the ability of the body to fight cancer, prevent its recurrence, and alleviate the side effects of conventional treatment (Ben-Arye et al., 2010; Wargovich, Woods, Hollis, & Zander, 2001). In fact, many medicinal herbs were experimentally shown to possess the above-mentioned activities in vitro and in vivo and may potentially improve cancer treatment outcomes.

Table 2 presents the evidence of biological activities pertinent to cancer treatment for 14 herbal medicines most commonly used by cancer patients. It also lists potential uses of these medicines in cancer therapy as well as possible side effects and drug interactions. As noted in Table 1, a number of herbs have demonstrated benefits in alleviating specific symptoms associated with cancer treatment or improving the overall quality of life. St. John’s wort and ginseng are effective in relieving stress, anxiety, and depression, symptoms commonly reported by cancer patients, whereas ginger helps control chemotherapy-induced nausea (Chang, Seo, Gyllenhaal, & Block, 2003; Levine et al., 2008; Manusirivithaya et al., 2004; Meyer, Schwartz, Crater, & Keyes, 1995; Nanthakomon & Pongrojpaw, 2006; Solomon, Ford, Adams, & Graves, 2011). St. John’s wort and black cohosh may help sleep by promoting relaxation and control of hot flashes, and herbal products such as echinacea, astragalus, and garlic stimulate the immune system and promote infection resistance (Block & Mead, 2003; Block, Gyllenhaal, & Mead, 2004; Hardy, 2008; Lai & Roy, 2004; Pockaj et al., 2004). Herbal use may also decrease chemotherapy and radiation toxicity, although the exact mechanism and effects of these powerful antioxidants remain unclear (Baatout et al., 2004; Hwang, Ha, & Park, 2005; Lev-Ari et al., 2005; Lev-Ari et al., 2007; Park et al., 2008; Premkumar, Thirunavukkarasu, Abraham, Santhiya, & Ramesh, 2006; Sadzuka, Sugiyama, & Hirota, 1998; Suganuma, Ohkura, Okabe, & Fujiki, 2001; Sugiyama & Sadzuka, 2004).

Some herbs have also shown the ability to directly affect the growth and spread of cancer or improve tumor response to conventional therapies (Hardy, 2008). They do so through a variety of mechanisms, including antioxidant protection of DNA, induction of apoptosis of cancer cells, stimulation of the immune system, and inhibition of cyclo-oxygenase, inflammation and angiogenesis (Ferrari, 2004). Finally, the third important potential benefit of herbal medicines for cancer patients is the prevention or postponement of cancer recurrence (Treasure, 2005; Wargovich et al., 2001), again achieved through the combination of antitumor, antioxidant, and immunomodulatory activities. Often the same biologically active compounds exhibit several of these activities, thus acting at multiple levels of cancer prevention and treatment at the same time.

**SAFETY CONCERNS**

In spite of all the purported benefits of herbal medicines in cancer, it is important to exercise caution when selecting and using herbal preparations during or after conventional cancer treatment. There is a belief among users of herbal medicines that these preparations are inherently safer than pharmaceuticals because they are "natural" and that they are effective over a wide range of doses. Because of these beliefs, patients often choose to self-medicate with herbal preparations, sometimes concurrently with conventional medications and without informing their physicians. This practice can be dangerous, especially for cancer patients. In fact, herbal products may contain multiple biologically active compounds, including some that have been isolated and are part of today’s pharmacologic treatment (Benzie & Wachtel-Galor, 2011). Just like conventional medications, herbal preparations can produce undesirable side effects such as allergic reactions, gastrointestinal symptoms, and shifts in blood values.

While the majority of the herbs reviewed in this article do not exhibit direct toxicity, others utilized by cancer patients have been found to be potentially harmful. For example, mistletoe (Iscador), used as an adjuvant cancer treatment to improve survival and overall quality of life (Kim, Yook, Eisenbraun, Kim, & Huber, 2012; Ostermann, Raak, & Büssing, 2009), can be hepatotoxic and should not be ingested (Harvey & Colin-Jones, 1981). Thus, mistletoe is administered by injection, which has an improved safety profile (Kim et al., 2012). Finally, the herbal medicines can interact with other medications taken by a patient. Rigorous scientific evaluation of the safety and effectiveness of herbal therapies, alone and with concurrent chemotherapy, as well as determination of optimal dosages and mechanisms of action are necessary before they can be recommended for cancer patients.

## USE OF VITAMINS, SUPPLEMENTS, AND OTHER NATURAL PRODUCTS

**BENEFITS AND RISKS**

As with herbs, there are innumerable products available to consumers in the United States, including vitamins, minerals, and many other natural compounds that can be used for self-care to prevent and treat illness, manage symptoms, improve mood, and enhance quality of life. These products (whether purchased online, at health food stores, and/or from conventional and CAM/integrative medicine providers) may include single substances or complex formulas of widely varying doses, which are created using different production methods that affect purity, potency, and efficacy. Safe, appropriate, and effective use therefore requires that consumers and health-care providers are informed and proactive in self-care strategies using natural products.

Vitamin and supplement use has varied widely over time, with noted fluctuations in popularity over the past decade. Data from the NHIS (2007) indicated that approximately 70% of the US cancer population (16.4 million) used multivitamins in the year preceding the interview. Whereas previous survey data (2002) examined use of megavitamins, this information was not queried in more current NHIS surveys. Popular vitamins and natural health supplements have included vitamins A, C, D, and E, calcium, magnesium, and zinc, fish oils/essential fatty acids (EFAs), a wide array of antioxidants for health promotion, and products for prevalent symptoms experienced by healthy individuals and those with chronic illness such as cancer. Since the number of vitamins and supplements is so vast, a discussion of selected products is presented in place of a review table. The authors acknowledge that this discussion is limited and refer the reader to the Natural Standard Database for a comprehensive review of natural health products (Natural Standard, 2013).

Products for cancer prevention and symptom management that were chosen for discussion include (a) vitamins A/beta-carotene, C, D, and E, selenium and lycopene to promote health and prevent cancer; (b) SAM-e and EFAs for mood concerns such as depression and anxiety; (c) melatonin, 5-HTP, and tryptophan to promote/improve sleep; (d) probiotics and glutamine for intestinal health and prevention and treatment of stomatitis and digestive problems; (e) glucosamine and EFAs for pain management; and (f) carnitine and CoQ10 for fatigue.

**PRODUCTS USED FOR CANCER PREVENTION**

Vitamins A/beta-carotene, C, and E, and selenium are antioxidants that have been studied extensively for cancer prevention and treatment effects, with conflicting findings. Use of high-dose vitamin A during cancer treatment remains highly controversial, with the possibility of toxicity. Beta-carotene supplementation has been noted to increase the risk of lung cancer among smokers (Goodman et al., 2004), although diets high in vitamin A have provided a protective lung effect in other studies (Lam, 2005). High intakes of vitamin A decreased the risk of esophageal cancer (Kubo & Corley, 2007), and when combined with vitamin C and E was beneficial for nonmelanoma skin cancer (Bath-Hextall, Leonardi-Bee, Smith, Meal, & Hubbard, 2007). Signs of toxicity include dry, itchy, scaling, and cracking skin, desquamation, dry lips, anorexia, headache, psychiatric changes, cerebral edema, bone and joint pain, and osteoporosis (Natural Standard, 2013).

Vitamin E, a fat-soluble vitamin that exists in eight different forms and may be natural or synthetic, has been studied for many different health conditions, including cancer and cardiovascular disease. Conflicting data about its anticoagulants (Table 3). According to the Natural Standard Database (2013), vitamins A, C, and E have unclear or conflicting scientific evidence (grade C), except for strong evidence (grade A) for vitamin A with regard to treatment of acute promyelocytic leukemia; selenium has also been graded as having good scientific evidence related to prostate cancer prevention (grade B).

**Table 3:**
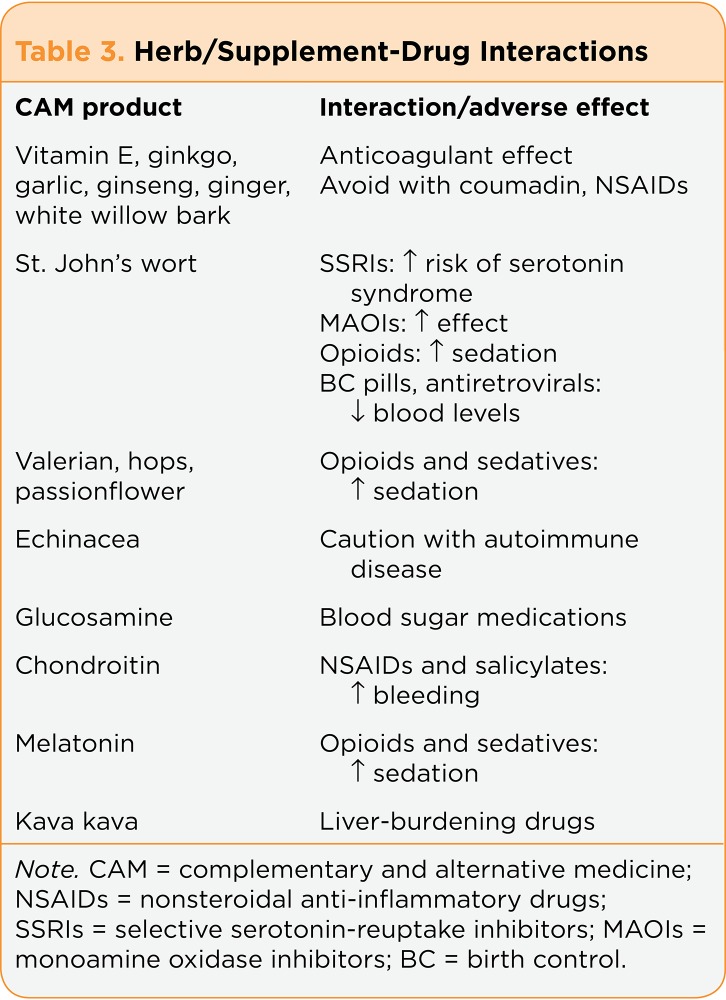
Table 3. Herb/Supplement-Drug Interactions

Vitamin D is critical to calcium and magnesium absorption in the body, thus protecting against osteoporosis, hypertension, some cancers, and autoimmune diseases. Vitamin D decreases the risk of colon cancer (Zeeb & Greinert, 2010) and mortality, although scientific evidence remains unclear and conflicting (Natural Standard, 2013). A recent systematic review also indicates that vitamin D supplementation decreases the need for analgesics in chronic pain sufferers (Straube, Derry, Moore, & McQuay, 2010). Of importance is the consideration of the type of vitamin D used and whether it is provided through food sources or as a supplement. Research has focused on vitamin D3, noting the toxicity of vitamin D2.

Lycopene, another powerful antioxidant, has received much attention lately because of study findings indicating a possible preventative effect in cancer and chronic illness. A natural substance found in foods such as tomatoes, lycopene supplements are available for over-the-counter purchase; however, most epidemiologic studies that indicate a reduced incidence of cancer, cardiovascular disease, and other disorders used food products rather than supplements. Studies have been conducted examining lycopene’s effects on prostate cancer, with level of evidence reported as unclear/conflicting at this point in time, indicating the need for rigorous controlled trials (Holzapfel et al., 2013; Kristal et al., 2011). Research is also needed to distinguish effects of lycopene found in food products as compared with lycopene supplementation on cancer prevention, treatment, and recurrence (Natural Standard, 2013).

**PRODUCTS USED FOR MOOD DISORDERS**

Essential fatty acids are polyunsaturated fatty acids that include eicosapentaenoic acid (EPA) and docasahexaenoic acid (DHA), which are found in marine/fish oils, and alpha-linolenic acid (ALA), found in plant oils such hemp and flaxseed. Essential fatty acids are considered anti-inflammatory supplements that support cardiovascular health; they are also used as mood stabilizers for individuals with attention deficit disorder, bipolar depression, seizures, mood imbalance, and psychotic disorders. Fish oil supplementation has also been found to increase response rate to chemotherapy in patients with advanced non–small cell lung cancer without increasing toxicity as compared with standard care, and it may increase survival (Murphy et al., 2011). Essential fatty acids may also reduce toxic side effects of some medications and were found to reduce pain associated with rheumatoid arthritis due to its anti-inflammatory effects (Ruggiero et al., 2009). Effects on cancer prevention remain unclear (MacLean et al., 2006; Sala-Vila & Calder, 2011), although essential fatty acids supplementation has improved appetite and cachexia in cancer patients (Colomer et al., 2007). Essential fatty acids have an anticoagulant effect and should be avoided with blood-thinning medications and herbs. Use of EFAs may also increase fasting blood sugar and the need for increased diabetic management medication (Natural Standard, 2013).

SAM-e (S-adenosyl-L-methionine), defined as a methyl group donor for many chemical reactions in the body, is primarily produced in the liver. SAM-e supplementation has been used for depression and psychiatric illness, premenstrual disorder, osteoarthritis, liver, and musculoskeletal problems, demonstrating antioxidant, anti-inflammatory, and analgesic effects (Natural Standard, 2013). Carney, Toone, and Reynolds (1987) noted that SAM-e crosses the blood-brain barrier and "is involved in several central enzyme pathways relating to transmethylation and folate and monoamine metabolism as well as in membrane function and neuro-transmission" (p. 104). Strong research evidence supports the use of SAM-e for analgesic effects in patients with osteoarthritis (Rutjes, Nüsch, Reichenbach, & Jüni, 2009). Adverse effects include nausea and diarrhea, and a risk of serotonin syndrome exists if used concurrently with certain groups of antidepressants (Iruela, Minquez, Merino, & Monedero, 1993). SAM-e is being explored in animal models to determine hepatoprotective effects and influence on chemotherapy (Ochoa, Bobadilla, Arrellín, & Herrera, 2009) and was found to have a preventative effect on FOLFOX (leucovorin, fluorouracil, and oxaliplatin)-induced liver toxicity (Vincenzi et al., 2011).

**PRODUCTS USED FOR SLEEP DISORDERS**

Melatonin, a neurohormone produced by the pineal gland, is involved in sleep regulation and continues to be studied for effects on tumor growth and regulation. Produced from the amino acid tryptophan, melatonin release is prompted by darkness and suppressed by light, indicating involvement in the body’s circadian rhythm (Brzezinski, 1997). Melatonin is rated as having good to strong scientific evidence (grades A and B) to support its use for jet lag, insomnia, and other sleep disorders in individuals of different ages (Natural Standard, 2013). Therapeutic benefits may also be related to melatonin’s antioxidant effects (Herzheimer & Petrie, 2002). At recommended doses, melatonin is regarded as safe for short-term use (Buscemi et al., 2005), although large doses have been used for longer periods. Side effects may include headaches, nausea, and dizziness; melatonin use may also decrease prothrombin time and has been implicated in seizures in children, although conflicting data have been reported (Sheldon, 1998; Wirtz, Spillmann, Bärtschi, Ehlert, & von Känel, 2008). Melatonin has been used orally as a supplement during cancer treatment as well as topically in studies on melanoma (Slominski et al., 2005). As an adjunct to cancer treatment, melatonin’s antioxidant properties may suppress the growth of cancer cells and increase natural killer (NK) cells that attack tumors (American Cancer Society, 2008). Recent research indicates its effectiveness against solid tumors and its cancer prevention potential (Grant, Melan, Latimer, & Witt-Enderby, 2009; Lissoni, Chilelli, Villa, Cerizza, & Tancini, 2003); a melatonin intervention for cancer treatment protocol is in progress (Wolf et al., 2012).

Tryptophan and 5-HTP (5-hydroxytryptophan) are supplements that are also used to promote sleep and improve sleep quality although scientific evidence is still unclear, with more studies needed. As a precursor to serotonin, 5-HTP has also been used for treatment of stress, anxiety, panic attacks, pain, depression, and obesity (Birdsall, 1998; Rothman, 2010; Weeks, 2009). Tryptophan, a precursor to 5-HTP, was recently allowed back on the US market after being banned for over a decade due to serious side effects (eosinophilia myalgia syndrome) that were subsequently attributed to a contaminant discovered in tryptophan supplements. 5-HTP is tolerated well at high doses although recommended use is 50 to 100 mg three times a day (Natural Standard, 2013). The level of evidence for obesity is rated as good (grade B) and unclear/conflicting (grade C) for anxiety, depression, fibromyalgia, and mood and sleep disorders (Natural Standard, 2013). Side effects include gastrointestinal discomfort and nausea, which are minimized by using enteric-coated capsules.

**PRODUCTS USED FOR GASTROINTESTINAL HEALTH**

Glutamine is a nonessential amino acid that continues to be examined for its preventative effects during cancer treatment, specifically with regard to mucositis. A recent systematic review of clinical trials indicated weak evidence from small studies where glutamine was used orally to prevent and promote healing of mucositis. Intravenous administration, however, appears to be beneficial for preventing severe mucositis, with additional studies needed (Worthington et al., 2011). Increased natural killer cell activity, inhibition of tumor growth (Klimberg et al., 1996), and prevention of chemotherapy-induced neuropathy in colorectal cancer patients has been documented with the use of glutamine (Wang et al., 2007). Glutamine may help stabilize weight loss during treatment by improving nutrient absorption, offering potential protection against cachexia. Also reported have been decreased hospital stays and medical expenses resulting from a reduced incidence of stomatitis and infection among patients undergoing cancer treatment (Anderson, Schroeder, & Skubitz, 1998; MacBurney, Young, Ziegler, & Wilmore, 1994). Although glutamine is generally considered to be nontoxic, large doses that may be used by cancer patients should be evaluated and closely monitored.

Probiotics and digestive enzymes are products that continue to be highly recommended within the natural health world and have also found their way into integrative medicine through use by naturopathic physicians, nurses, advanced practitioners, chiropractors, and others providing patient care. Probiotics are beneficial bacteria/microorganisms found in foods and also available as supplements that are used for the prevention and treatment of illness. Strong research evidence supports their use for gastroenteritis and management of acute diarrhea, a serious worldwide problem threatening survival, especially among children and the elderly (WHO, 2001). There is also evidence supporting safety of their use for allergies, urinary tract infections, Crohn’s disease, irritable bowel syndrome, and Clostridium difficile colitis. Animal studies have indicated ability of probiotics to hinder absorption of aflatoxins implicated in liver cancer and inhibition of colon and bladder cancers (Goldin & Gorbach, 2008).

Digestive enzymes are naturally occurring biological molecules secreted by different glands (such as the salivary glands, stomach and pancreas) that assist with the breakdown of food into usable substances to facilitate absorption of nutrients that sustain life. Types of digestive enzymes include (a) protease to break down proteins into peptides and amino acids; (b) lipase to break down fats into fatty acids; and (c) carbohydrases, such as amylase, cellulase, and lactase, to break down carbohydrates into starch and sugars.

Supplemental digestive enzymes have been used to maintain health and prevent illness, as well as an adjunctive treatment for diseases such as cancer. The most notable has been the use of pancreatic enzymes and nutritional therapy for advanced-stage pancreatic cancer using the Gonzalez regimen, which remains under study in phase III trials funded by the National Institutes of Health/National Cancer Institute. This regimen was developed by Nicholas Gonzalez in the 1990s and is based on the work of William Kelley and Max Gerson, physicians who treated cancer patients with diet and supplements. The protocol is also based on the idea that pancreatic enzymes could kill cancer cells, a theory advanced by John Beard in the early 1900s. The Gonzalez protocol includes the use of orally administered pancreatic enzymes, high doses (130–160/day) of nutritional supplements, a special diet, and coffee enemas. This cancer treatment protocol aims to rid the body of environmental toxins, promote immune system function, and balance the nervous system, strategies that are thought to fight cancer (National Cancer Institute, 2013).

Digestive enzymes are available over the counter for self-treatment and through CAM practitioners using specified protocols with high doses for cancer treatment. Specified and safe dosing has not been established (Natural Standard, 2013). A potential protective effect of proteolytic enzymes on radiation therapy side effects has been reported (Dale, Tamhankar, George, & Daftary, 2001; Gujral et al., 2001; Sakalová et al., 2001) as well as a decrease in mortality among patients with multiple myeloma with use of oral enzyme therapy (Sakalová et al., 2001).

**PRODUCTS USED FOR PAIN**

There are many CAM products that are being used for the management of pain, which remains a highly prevalent problem in the United States. They include supplements such as glucosamine and chondroitin for osteoarthritis (OA), taken as an oral or topical supplement. Over 2 decades of intense research has yielded positive results, although some inconsistency is noted, requiring longitudinal studies, which are in progress. Strong evidence exists that glucosamine does improve pain related to knee OA (grade A) and OA in general (grade B; Natural Standard, 2013) with reduction in the use of nonsteroidal anti-inflammatory agents. Improvement in joint-space narrowing has also been reported (Fouladbakhsh, 2012). Since cancer prevalence is highest in older adults, many also experience pain related to OA and other comorbidities, and may use glucosamine with or without chondroitin. Glucosamine has also been related to a reduced risk of death from cancer and respiratory disease (Bell, Kantor, Lampe, Shen, & White, 2012).

**PRODUCTS USED FOR FATIGUE**

Fatigue remains one of the most prevalent and burdensome problems during cancer treatment and the survivorship period, with treatments including nutritionally sound diets, exercise programs, multivitamins, medications and other supplements, acupuncture, and mind-body therapies (Sood, Barton, Bauer, & Loprinzi, 2007).

Ginseng. A recent study at the Mayo Clinic found that 8 weeks of ginseng (2,000 mg American ginseng per day) resulted in significant improvement in general exhaustion in the cancer group as compared to the placebo group. A "20% improvement in fatigue in cancer patients, measured on a 100-point, standardized fatigue scale" was noted by lead researcher Debra Barton (2012). The researchers further stressed that ginseng, used for thousands of years in traditional Chinese medicine, helps to reduce proinflammatory cytokines and cortisol, which are elevated in chronic fatigue and stress, respectively, in cancer patients and survivors.

Carnitine, specifically referred to as levocarnitine (L-carnitine) is a "carrier molecule involved in the transport of long-chain fatty acids across the inner mitochondrial membrane and is required in mammalian energy metabolism" (Sood et al., 2007, p. 10). Since carnitine is involved in metabolism, it has been examined as a potential supplement to improve fatigue.

Coenzyme Q10, also known as ubiquinone, is a fat-soluble antioxidant that is under study for its effects on cardiovascular health, Alzhiemer’s disease, cancer, and other health conditions. Produced by the body, it is involved in the generation of adenosine triphosphate (ATP), and hence has been examined in relationship to energy production and antioxidant effects (Niklowitz, Sonnenschein, Janetzky, Andler, & Menke, 2007). A recent study among breast cancer patients has indicated that although supplementation raised CoQ10 blood levels, no improvement in self-reported fatigue or quality of life was noted as compared to the control group (Lesser et al., 2013). Continuing studies are underway at the National Institutes of Health/National Cancer Institute to determine the effects of supplemental CoQ10 on chronic illness and symptom management (www.clinicaltrials.gov).

In sum, all herbal medicines and supplements reviewed in this article possess biological activities that may be of great benefit to cancer patients, either by enhancing the positive effects of conventional therapies, while alleviating their undesirable side effects, or by preventing the recurrence of cancer. However, not enough is known at present about the exact composition of many herbal therapies and other CAM natural products, their mechanism of action, and safe and effective dosages. For this reason, it is often recommended that patients avoid the use of herbal preparations and supplements before and after surgeries and during active chemotherapy and radiation until further research is conclusive. It is also of utmost importance that patients inform their health care providers of their use of herbs, vitamins, and supplements to maximize safety and treatment effectiveness while minimizing the risks of potential interactions.

## ROLE OF THE ADVANCED PRACTITIONER

Given the potential benefits, as well as risks, associated with the use of natural health products for the prevention and treatment of cancer, and the amelioration of the side effects of conventional cancer treatment, it is imperative that advanced practitioners acknowledge natural products within their oncology practice (Balneaves, Weeks, & Seely, 2008). Foremost, it is within the scope of practice of advanced practitioners to assess cancer patients and survivors for use of, and interest in, CAM therapies, including natural health products. Asking about the use of herbal therapies and vitamin and mineral supplements in a nonjudgmental manner is the first step to opening the dialogue about natural health products and determining cancer patients and survivors’ information and decision support needs. Guidelines to support effective communication about CAM therapies in oncology settings exist (Schofield, Diggens, Charleson, Marigliani, & Jefford, 2010) and provide advanced practitioners with direction regarding how to respectfully address natural product use.

The complex and uncertain nature of much of the evidence surrounding natural health products often leaves cancer patients and survivors confused and anxious about making an informed decision. Advanced practitioners are well positioned to engage cancer patients and survivors in a shared decision-making process, in which the available evidence about natural health products is balanced against the values, beliefs, and personal context of the individual. Shared decision-making is a theoretical model (Charles, Gafni, & Whelan, 1997) that supports collaboration between patients and practitioners in treatment decisions in discussing the best available evidence and the benefits, risks, and uncertainties associated with each therapy option.

This approach to decision making is respectful of patient autonomy and preferences and has been recently put forward as an "ethical imperative" in clinical care (Salzburg Global Seminar, 2011). In the context of natural health products, shared decision-making requires advanced practitioners to be familiar with evidence-based information resources and informed about the latest evidence associated with popular products and supplements (Balneaves et al., 2012). With the growing prevalence of natural health product use in cancer populations, this knowledge has become an imperative if advanced practitioners are to provide care and support that are evidence-based and comprehensive (Bauer-Wu & Decker, 2012).

The FIRST Approach to CAM©, developed in integrative nursing practice, provides a framework to understand and guide CAM use among patients (Table 4; Fouladbakhsh, 2013). In this approach, health-care providers must take the initiative to become familiar with CAM therapies and learn about CAM natural health products by seeking current and accurate information about effects, safety, and research evidence. We must also have knowledge of the right providers, understanding the education and credentialing processes and how to locate trained and certified CAM practitioners. Inherent in our practice, we must also focus on our own self-care, a fundamental principle within holistic nursing practice, which keeps us healthy, grounded and available to patients who seek our assistance. Only then can we understand and effectively support their self-care initiatives. Last, but certainly not least, we must encourage patients to tell their doctors and other health-care providers about their CAM use. This will help prevent interactions while promoting safe and beneficial outcomes. Using the FIRST approach can promote appropriate, informed and safe use of CAM products as patients seek relief and hope during their cancer journey using integrative approaches to their care.

**Table 4:**
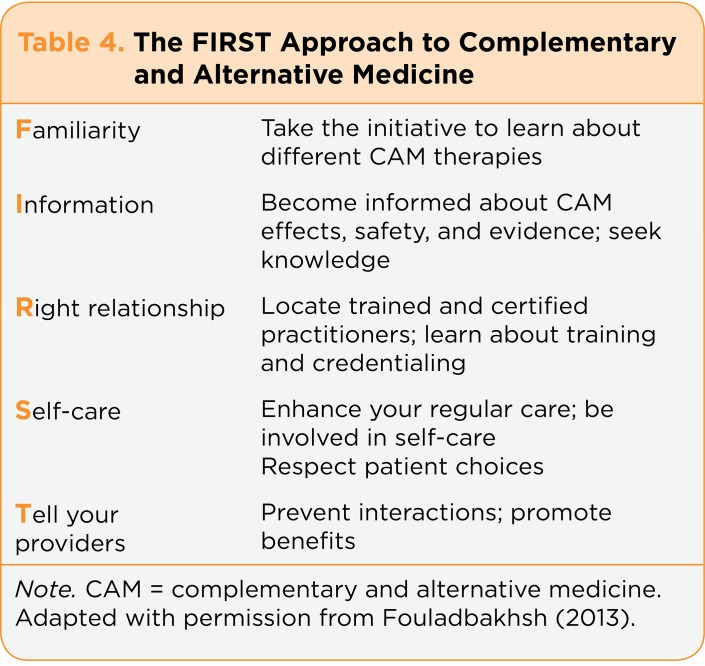
Table 4. The FIRST Approach to Complementary and Alternative Medicine

## CONCLUSIONS

It is critical that advanced practitioners begin by assessing patient use of CAM therapies at the time of diagnosis and be prepared for the answers they receive. This will prevent deleterious side effects and interactions that may negatively affect the treatment and recovery process. To date, much uncertainty remains regarding the interactive effects of herbs and natural products on the CYP450 and other metabolic pathways, and the influence on drug levels, tumor response, and elimination of toxins. Continued research is needed to more fully illuminate these effects, providing clear guidelines on use and potential benefits that may exist for cancer patients. Only then will we as health-care professionals have a clear understanding of the impact of CAM products on the lives of our patients.
